# Transforming growth factor-β suppresses metastasis in a subset of human colon carcinoma cells

**DOI:** 10.1186/1471-2407-12-221

**Published:** 2012-06-06

**Authors:** Neka A K Simms, Ashwani Rajput, Elizabeth A Sharratt, Melanie Ongchin, Carol A Teggart, Jing Wang, Michael G Brattain

**Affiliations:** 1 Eppley Institute for Research in Cancer and Allied Diseases, University at Nebraska Medical Center, Omaha, USA; 2 Department of Surgery, Division of Surgical Oncology, University of New Mexico, Albuquerque, USA; 3 Department of Pharmacology and Therapeutics, Roswell Park Cancer Institute, Buffalo, USA; 4 Department of Surgery, University at Buffalo, Buffalo, USA

## Abstract

**Background:**

TGFβ signaling has typically been associated with suppression of tumor initiation while the role it plays in metastasis is generally associated with progression of malignancy. However, we present evidence here for an anti-metastatic role of TGFβ signaling.

**Methods:**

To test the importance of TGFβ signaling to cell survival and metastasis we compared human colon carcinoma cell lines that are either non-tumorigenic with TGFβ response (FET), or tumorigenic with TGFβ response (FETα) or tumorigenic with abrogated TGFβ response via introduction of dominant negative TGFβRII (FETα/DN) and their ability to metastasize. Metastatic competency was assessed by orthotopic transplantation. Metastatic colony formation was assessed histologically and by imaging.

**Results:**

Abrogation of TGFβ signaling through introduction of a dominant negative TGFβ receptor II (TGFβRII) in non-metastatic FETα human colon cancer cells permits metastasis to distal organs, but importantly does not reduce invasive behavior at the primary site. Loss of TGFβ signaling in FETα-DN cells generated enhanced cell survival capabilities in response to cellular stress *in vitro*. We show that enhanced cellular survival is associated with increased AKT phosphorylation and cytoplasmic expression of inhibitor of apoptosis (IAP) family members (survivin and XIAP) that elicit a cytoprotective effect through inhibition of caspases in response to stress. To confirm that TGFβ signaling is a metastasis suppressor, we rescued TGFβ signaling in CBS metastatic colon cancer cells that had lost TGFβ receptor expression due to epigenetic repression. Restoration of TGFβ signaling resulted in the inhibition of metastatic colony formation in distal organs by these cells. These results indicate that TGFβ signaling has an important role in the suppression of metastatic potential in tumors that have already progressed to the stage of an invasive carcinoma.

**Conclusions:**

The observations presented here indicate a metastasis suppressor role for TGFβ signaling in human colon cancer cells. This raises the concern that therapies targeting inhibition of TGFβ signaling may be imprudent in some patient populations with residual TGFβ tumor suppressor activity.

## Background

Metastatic disease accounts for 90% of cancer related deaths in all cancers [1]. The metastatic process requires the ability of the tumor to invade at the primary site, undergo intravasation, survive immune surveillance in blood circulation, undergo extravasation at a distal organ site and form new colonies at this secondary organ site [2]. Molecular mechanisms involved in the establishment of metastases are largely unknown. Understanding molecular mechanisms involved in the metastatic process could identify novel potential targets for development of more effective therapeutic intervention against established metastatic disease.

An important aspect of metastatic potential is the ability of a cancer cell to evade apoptotic signals under stress conditions which could normally lead to cell death [3,4]. Evasion of apoptosis can occur as a result of loss of tumor suppressor activity and/or enhanced oncogenic activity thus shifting the balance of stress response toward inappropriate cell survival. Many cellular pathways have been linked to enhanced survival or anti-apoptotic signaling and malignant progression; here we investigated the role of transforming growth factor-β (TGFβ) in an orthotopic colorectal cancer model of metastasis.

The general consensus is that TGFβ signaling is tumor suppressive in early carcinogenesis, but it becomes tumor promoting during later stages of cancer [5]. TGFβ signaling through Smad activation is regarded as tumor suppressive during the early stages of cancer and pre-cancerous lesions as it has been shown that loss of TGFβ tumor suppressor signaling has been associated with tumor initiation and progression of several types of tumors including colon cancer. TGFβRII has been shown to be inactivated by mutation in human colon cancers with microsatellite instability [6]. Other types of cancer as well as some subsets of colon cancer are often associated with epigenetic transcriptional repression of TGFβ receptors rather than mutational inactivation of the pathway [7-9], ultimately contributing to a loss in growth control as well as resistance to apoptosis [10,11]. Studies conducted in breast cancer demonstrated that the unmodified transcription factor Sp3 induces transcriptional repression of TGFβRII promoter [7]; consequently, treatment with histone deacetylase inhibitor, Trichostatin A (TSA), results in acetylated Sp3 which alleviates transcriptional repression of TGFβRII gene expression [8]. On the other hand, it has been reported that increased expression of receptor ligands by tumor cells was associated with tumor progression in non-small cell lung cancer (NSCLC), colorectal cancer and gastric carcinomas [12-14]. Thus, one view is that TGFβ tumor promotion may occur predominantly in situations where signaling receptor expression is deficient [15].

Loss of TGFβ tumor suppressor signaling is important in a tumor cell’s ability to evade apoptotic signaling in the tumor microenvironment. Previously, our laboratory identified the linkage of TGFβ tumor suppressor activity to the repression of pro-survival PI3K/AKT signaling and linked the PI3K/AKT pathway to survivin expression in human colon carcinoma cell lines [16]. AKT has a wide variety of substrates involved in many cellular responses including proliferation, apoptosis and growth. Over-expression and/or constitutive signaling of PI3K/AKT pathway components have frequently been implicated in the regulation of cell survival and their association with tumor progression [17].

Survivin, also known as Birc5, is a 16.5 kDa protein that is the smallest member of the inhibitors of apoptosis (IAP) family. Survivin is expressed in the nucleus, the cytosol and the mitochondria. Survivin is expressed in proliferating cells such as embryonic and fetal cells and is undetectable in differentiated normal tissue; however, survivin is highly expressed in numerous solid tumor types including colon, breast, lung and liver, and its expression is associated with aberrant cell survival and tumor progression [18-20]. Overexpression of survivin has been associated with inhibition of cell death initiated by extrinsic or intrinsic apoptotic pathways [21]. Survivin expression is associated with poor clinical prognosis in many tumor types including colon, lung and breast [22-25]. Survivin protects X-linked inhibitor of apoptosis (XIAP) from proteasomal degradation and antagonizes apoptosome-mediated cell death through the ability of XIAP to inhibit caspase activation [26]. It has been shown that upon cellular stress, mitochondrial survivin is released into the cytosol where it interacts and stabilizes XIAP and provides protection from cell death [27]. The Bir2 domain of XIAP has been linked with inhibition of caspase 3 and caspase 7; and the Bir3 domain with caspase 9 inhibition [28]. AKT/PKB-mediated phosphorylation of XIAP within the Bir1 domain is implicated in reducing auto-ubiquitination and enhanced protein stabilization [29].

Many studies indicate that aberrant TGFα/EGFR signaling is involved in tumor progression [30-34]. The FET colon cancer cell line which normally does not form subcutaneous xenografts in athymic mice [35] becomes highly tumorigenic after TGFα (transforming growth factor-α) transfection to generate constitutive EGFR (epidermal growth factor receptor) activation [36]. FET cells have robust autocrine TGFβ signaling that inhibits cell proliferation and contributes to apoptosis in response to stress [16]. We show here that FETα cells exhibit robust invasion at the primary site after orthotopic implantation. The ability to invade at the primary site is the key attribute in the assignment of cancer diagnosis [37]. Importantly, however, despite invasive capabilities, the FETα cells rarely metastasize when implanted at the orthotopic site of the colon in athymic mice. Ye et al. [38] demonstrated that repression of TGFβ activity by transfection of dominant negative (DN) TGFβRII was sufficient to lead to vigorous tumor growth by FET cells in subcutaneous implants; however, as with FETα cell induced tumors FETDNRII orthotopic implants without ectopic TGFα expression resulted in invasive primary cancers that rarely metastasized. Since the TGFβ receptor/SMAD signaling in FETα cells remained intact, we hypothesized that suppression of this pathway would be sufficient to generate a metastatic phenotype in association with increased resistance to apoptosis in response to stress from orthotopic transplants. Two mechanisms contributing to increased survival associated with loss of TGFβ tumor suppressor activity are constitutive AKT activation and survivin/XIAP expression. These results show that in addition to suppression of tumor initiation, TGFβ signaling provides a direct mechanism of metastatic suppression in established carcinomas. To substantiate our findings that TGFβ signaling is a metastatic suppressor in established carcinomas, we utilized a human colon carcinoma cell line (designated CBS) that is metastatic after orthotopic implantation and demonstrates loss of TGFβ signaling due to epigenetic repression of the TGFβRII. Ectopic expression of TGFβRII in CBS-RII cells resulted in primary carcinoma formation as reflected by invasion, but was accompanied by suppression of the metastatic phenotype in the orthotopic implantation model. Also, reintroduction of Smad-dependent TGFβ signaling resulted in decreased expression of cytoplasmic survivin and XIAP in CBS-RII cells. Taken together, our results suggest that restoration of TGFβ signaling in non-responsive metastatic cells can inhibit cell survival and metastases. Moreover, the role of TGFβ receptor/Smad signaling in curtailing metastatic progression in primary invasive carcinoma suggests that strategies involving inhibition of TGFβ signaling for cancer treatment may be ill-advised for some subpopulations of cancer patients.

## Methods

### Cell lines and reagents

FETα and FETα-DN colon carcinoma cells were cultured at 37°C in a humidified atmosphere of 5% CO_2_ in SM medium [McCoy’s 5A serum-free medium (Sigma) with pyruvate, vitamins, amino acids, and antibiotics] supplemented with 10 ng/mL EGF, 20 μg/mL insulin, and 4 μg/mL transferrin. When the cells were subjected to growth factor deprivation stress (GFDS), they were cultured in SM medium in the absence of growth factor or serum supplements for 24 or 48 h without medium changes in between. Antibodies for poly (ADP-ribose) polymerase (PARP), AKT, phosphorylated AKT (Ser473), and survivin were obtained from Cell Signaling Technology. Actin and tubulin antibodies were purchased from Sigma. P-Smad2 and XIAP antibodies were from Chemicon and Abcam, respectively. PI3K inhibitor LY294002, and TGFβ were obtained from Calbiochem. Apoptag plus Peroxidase *In Situ* Apoptosis Detection kit was from Millipore/Chemicon and both the DAKO Envision System HRP and the monoclonal anti-Human KI-67 antigen (Clone Mib-1) were from DAKO North America. Annexin V-FITC Apoptosis Detection kit (including propidium iodide) was from BD Bioscience Pharmingen while the Cell Death Detection ELISA^PLUS^ kit was from Roche Diagnostics. Hematoxylin was obtained from Protocol and eosin was from Sigma-Aldrich.

### Ectopic expression of dominant negative TGFβRII receptor

The DNRII expression vector was described previously [38]. The cDNA was subcloned into a MX-IV retroviral vector. The 293GP packaging cells (Clontech, Mountain View, CA) were co-transfected with pVSV-G. The viruses were harvested 48 h later and used to infect FETα cells. Puromycin (3.0 μg/mL) was used to select infected cells for 8 days and then cells were pooled.

### Immunoblot analysis

Cells were lysed in TNESV lysis buffer [50 mmol/L Tris (pH 7.5), 150 mmol/L NaCl, 1% NP40, 50 mmol/L NaF, 1 mmol/L Na_3_VO_4_, 25 μg/mL h-glycerophosphate, 1 mmol/L phenylmethylsulfonyl fluoride, one protease inhibitor cocktail tablet (Roche, Indianapolis, IN) per 10 mL] for 30 minutes on ice. The supernatants were then collected by centrifugation at 21,000×g for 15 minutes at 4°C. Protein was determined by the Pierce BSA method. Proteins samples were dissolved in 1× sample buffer (50 mM Tris, pH6.8, 1% SDS, 10% glycerol, 0.03% bromophenol blue and 1% β-mercaptoethanol). Protein (10–50 μg) was fractionated on a 10% acrylamide denaturing gel and transferred onto a nitrocellulose membrane (Life Science, Amersham) by electroblotting. The membrane was blocked with 5% nonfat dry milk in TBST [50 mmol/L Tris (pH 7.5), 150 mmol/L NaCl, 0.05% Tween 20] for 1 h at room temperature or overnight at 4°C and washed in TBST. The membrane was then incubated with primary antibodies at 1:1000 dilutions for 1 h at room temperature or overnight at 4°C. After washing with TBST for 30 min, the membranes were then incubated with peroxidase-conjugated goat anti-mouse or anti-rabbit IgG (Jackson ImmunoResearch Laboratories, Inc) at a 1:1,000 dilution for 1 h at room temperature. After further washing in TBST for 30 min, the proteins were detected by the enhanced chemiluminescence (ECL) system (Amersham) or SuperSignal West Pico Chemiluminescent Substrate (Thermo Scientific).

### Immunoprecipitation

Cells were lysed in TNESV lysis buffer for 30 minutes on ice. The supernatants were then collected by centrifugation at 21,000×g for 15 minutes at 4°C. Protein was determined by the Pierce BSA method. Protein (300 ug) was pre-cleared with 10ul of protein A/G beads and lysis buffer for 30 minutes at 4°C. Samples were centrifuged at 21,000 × g at 4°C for 10 minutes followed by collection of the supernatant. The supernatant was incubated while rotating with antibody (according to the manufacturer’s specifications) at 4°C for 60 minutes, followed by addition of 25 ul protein A/G beads and tumbled overnight. Samples were centrifuged at 21,000 × g for 1 minute at 4°C. The supernatant was collected to probe for actin as an experimental control, while the pellet was washed 3 times for 5 minutes in lysis buffer at 21,000 × g at 4°C, each time the supernatant was decanted. The pellets were dissolved in 20 ul 1x sample buffer (50 mM Tris, pH6.8, 1% SDS, 10% glycerol, 0.03% bromophenol blue and 1% β-mercaptoethanol) and boiled for 5 minutes at 95°C, then spun and loaded on SDS-PAGE gel.

### DNA fragmentation (cell death ELISA)

Apoptosis was quantified by a DNA fragmentation ELISA. Briefly, cells were seeded in plates in serum-free medium and allowed to attach for 24 hours. Medium was changed on alternate days until 80% confluence was attained. Next, the medium was changed to supplemental McCoys for 24 or 48 h of growth factor deprivation stress (GFDS). DNA fragmentation was detected by the Cell Death Detection ELISA Plus kit (Roche, Indianapolis, IN) according to the manufacturer’s instructions. DNA fragmentation was normalized by MTT assays derived at identical treatment conditions.

### MTT (3-(4, 5-dimethylthiazol-2-yl)-2, 5-diphenyltetrazolium bromide)

Cells were grown to 80% confluence then MTT was added to the medium followed by incubation at 37°C for 2 h. The medium was aspirated to visualize stained cells. DMSO was added and the plate was covered with foil followed by shaking for 15 min. Duplicates volumes (150 μL) were added to a 96-well plate and the absorbance was observed at 570 nm.

### [^3^H] Thymidine incorporation

[^3^ H] Thymidine incorporation was used to determine cell cycle inhibition of FETα and FETαDN cells after TGF-β treatment. The cells were seeded in six-well tissue culture plates and grown to 60% confluence. At 48 h after TGFβ treatment, the cells were labeled with [^3^ H] thymidine (7 μCi; 46 Ci/mmol; Amersham Corp.) for 1 h. DNA was then precipitated with 10% trichloroacetic acid and solubilized in 0.2 mol/L NaOH. The amount of [^3^ H] thymidine incorporated was analyzed by liquid scintillation counting in a Beckman LS7500 scintillation counter.

### Immunohistochemistry (IHC)

Primary tumors established from the FETα and FETα-DN cells were harvested and placed in 10% neutral buffered formalin fixative for 12 to 24 hrs and then embedded in paraffin. Deparaffinized tissue specimens were subjected to immunohistochemical staining for the detection of pAKT-S473, survivin and XIAP using an indirect detection method [39]. The catalyzed signal amplification system was used for the phosphospecific antibodies (Dako Corporation, Carpinteria, CA). The antibody staining was accompanied by a negative control in which slides were incubated with a matching blocking peptide (Dako Corporation) to the primary antibody. Specimens were processed on the same day to eliminate any variability in conditions. Slides were digitally photographed using the same settings.

### Terminal deoxynucleotidyl transferase-mediated dUTP nick end labeling (TUNEL) assay

Slides were cut from paraffin embedded blocks and stained according to the Apotag (Oncor, Gaithersburg, MD) terminal nucleotidyl transferase–mediated nick end labeling (TUNEL) assay kit. The apoptotic rate was quantitatively determined by counting the number of positively stained apoptotic bodies per 75 μm^2^ field at 200x magnification. Twelve and fifteen histological slides for the FETα and FETα-DN tumors, respectively, were analyzed. Three histologically similar fields viewed at 200X were randomly selected from each slide for analysis. This procedure was performed by two blinded observers. The ratio of the average number of apoptotic cells to the total number of cells counted was used to represent apoptotic rates.

### KI-67 staining

Slides cut from paraffin embedded blocks were also used for H&E stains and for immunohistochemical characterizations. Serial sections were cut to complement the H&E sections and were stained with an IgG_1_ rabbit polyclonal antibody for Ki-67 (Dako Corporation). Ki-67 is a non-histone nuclear antigen present in late G_1_, G_2_, and S phase of the cell cycle but not G_0_. The optimal dilution of 1:100 was used. Three- to 4-μm sections were cut, deparaffinized in xylene, and rehydrated in descending grades of ethanol. Endogenous peroxidase activity was blocked with 3% hydrogen peroxide in water. Immunostaining was done using a variation of the avidin-biotin-peroxidase method. Slides were counterstained with methyl green. The proliferation rate was determined quantitatively by utilization of NIH Image J (public domain software). Image settings were as follows: threshold range 10–192; pixel size 20–5000. Twelve slides from FETα and FETα-DN were analyzed. Three histologically similar fields viewed at 200X were randomly selected for analysis. The mean proliferation was determined for each group.

### Subcellular fractionation

Cells were washed with phosphate buffered saline (PBS) then lysed using 500 μl of fractionation buffer (250 mM Sucrose, 20 mM HEPES pH7.4, 0 mM KCl, 1.5 mM MgCl_2_, 1 mM EDTA, mM EGTA). Cells were scraped immediately and placed in a 1.5 ml eppendorf tube on ice. Collected cells were then passed through a 25 G needle 10 times, and incubated on ice for 20 min. Cells were centrifuged at 720 × g (360 rpm) for 5 min to isolate the nuclear pellet from the supernatant containing the cytoplasm. The nuclear pellet was washed with fractionation buffer and passed through a 25 G needle 10 times followed by centrifugation at 720 × g (360 rpm) for 10 min again. The supernatant containing the cytoplasm was centrifuged at 14,000 × g (8000 rpm) for 10 min to yield the cytosolic fraction (supernatant) and the mitochondrial fraction (the pellet). The mitochondrial pellet was washed with fractionation buffer and passed through a 25 G needle 10 times followed by centrifugation at 14,000 × g (8000 rpm) for 10 min again then re-suspended in the appropriate volume of fractionation buffer.

### Orthotopic implantation

Orthotopic implantation was performed as previously described [40]. Briefly, green fluorescent protein (GFP)-labeled FETα and FETα-DN cells (5 × 10^6^) were subcutaneously injected onto the dorsal surfaces of separate BALB/c nude male mice and allowed to grow to 300 mm^3^. Once xenografts were established, they were excised and minced into 1 mm^3^ pieces. Two of these pieces were then orthotopically implanted into the colon of other BALB/c nude mice. Forty four animals were implanted with FETα xenografts and 30 animals with FETα-DN xenografts. For operative procedures, animals were anesthetized with isoflurane inhalation. A 1-cm laparotomy was performed and the cecum and ascending colon were exteriorized. Using 7X magnification and microsurgical techniques, the serosa was disrupted in two locations. Pieces of xenograft (1 mm^3^) were subserosally implanted using an 8–0 nylon suture at the disrupted serosal locations. The bowel was then returned to the peritoneal cavity and the abdomen was closed with 5–0 vicryl suture. Fluorescence imaging was performed weekly on the animals to follow tumor growth (LightTools). Animals were euthanized at 7–9 weeks after implantation. Organs were explanted, imaged, and immediately placed in buffered 10% formalin. Tissues were then processed and embedded in paraffin. Histological slides were cut for H&E staining. Metastases were determined by histological evaluation of each liver lobe and both lungs as previously described in detail [41,42]. All animal work was done in accordance with the Institutional Animal Care and Use Committees (IACUC) regulations. Protocol number was 920 M.

### Imaging

Starting at one week post-implantation, animals were anesthetized with a 1:1 mixture of ketamine (10 mg/ml) and xylazine (1 mg/ml) by intraperitoneal injection (0.01 ml/mg) and weekly GFP fluorescence imaging was performed for up to seven weeks. Specifically, GFP fluorescence imaging was performed using a light box illuminated by fiber optic lighting at 470 nm (Illumatool BLS, Lightools Research, Encinitas, CA) using a Retiga EXi color CCD camera (QImaging, Burnaby BC, Canada). High-resolution images consisting of 1,360 X 1036 pixels were captured directly using a MS-Windows based PC. Images were visually optimized for contrast and brightness using commercial software (Adobe Photoshop, CS2 Adobe, San Jose, CA). Excitation of GFP in the light box facilitated identification of primary and metastatic disease by direct near-real time visualization of fluorescence in live animals.

## Results

### TGFβ suppresses metastasis *in vivo*

We have reported that the FET cell line which was isolated from a human colon cancer is immortalized and grows with anchorage independence, but does not form tumors in athymic mice after subcutaneous implantation [32]. Stable transfection with a construct coding for active (processed) TGFα under TET off control resulted in progressive growth at the subcutaneous site in the absence of TET. With the addition of TET the FETα tumors showed regression in association with high apoptotic rates as reflected by TUNEL [33]. FETα cells as well as the parental FET cell line have a high sensitivity to TGFβ in contrast to most cancer derived cell lines. We hypothesized that TGFβ signaling suppresses metastasis of FETα cells. To test this hypothesis, we stably co-transfected FETα cells with a dominant negative RII receptor construct and denoted these cells as FETα-DN. Abrogation of TGFβ signaling was confirmed by treating FETα and FETα-DN cells with varying concentrations of TGF β [0, 5, 10 ng/mL] for 2 h followed by immunoblot analysis. Phospho-Smad2 was used as an indicator of functional TGFβ signaling. FETα cells showed a concentration-dependent induction of pSmad2 while FETα-DN cells showed no pSmad2 expression. This result confirmed loss of TGFβ receptor mediated Smad signaling in FETα-DN (see Additional file 1).

**Figure 1:**
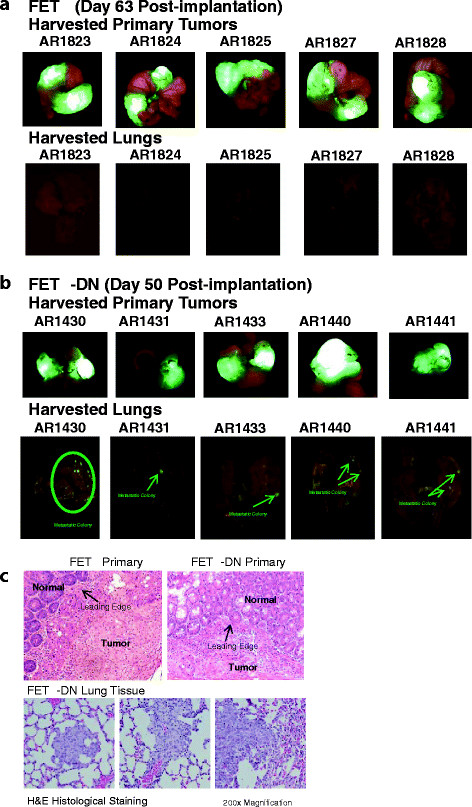
**Orthotopic implantation of xenografts formed by FETα and FETα-DN cells are shown. (A)** Fluorescent images of isolated primary tumors and lungs from FETα orthotopically implanted animals. Green fluorescence reflects colony formation. Note the lack of fluorescent nodules. **(B)** Fluorescent images of isolated primary tumors and lungs from FETα-DN orthotopically implanted animals. Green fluorescence reflects colony formation. **(C)** Primary tumors from FETα and FETα-DN orthotopic implants highlighting the leading edge of invasion (top); and of metastases in the lungs of orthotopic FETα-DN implanted animals with H&E staining. Images were captured at 200x magnification.

Comparison of FETα and FETα-DN cells by orthotopic implantation was used to assess the effect of loss of TGFβ receptor/Smad signaling on malignant progression beyond the first step of the metastatic process (invasion of the primary tumor) as reflected by metastatic colonization of distant organs from the primary tumor site. Forty four animals were implanted with FETα cells and 30 animals with FETα-DN cells. Metastatic spread was analyzed in liver and/or lungs of transplanted mice as described in the methods. The presence or absence of metastatic disease was determined by microscopic histological analysis of lungs and liver from mice bearing orthotopic implants as previously described [42]. We observed 100% primary tumor growth and invasion at the primary site of implantation for all animals, however only 2/44 animals showed metastatic colony formation in the lungs or liver from orthotopic implantation of FETα cells (Table 1). Figure 1A shows images of FETα implants with GFP fluorescence of isolated primary tumor tissue and lungs with no visible GFP fluorescence. Table 2 summarizes the results of orthotopic implantation with subcutaneous xenografts formed by injection of FETα-DN cells. As with FETα orthotopic implants we observed 100% primary tumor growth and invasion at the primary site of implantation for all animals: in addition, visible GFP fluorescence from metastatic cells was evident in the lungs. The results show that 23/30 animals from FETα-DN bearing animals had metastatic colony formation in the lungs. Figure 1B shows images of FETα-DN implants with GFP fluorescence by primary tumor tissue and lungs with visible deposits of GFP fluorescent colonies. Metastatic colonization was also evaluated histologically by hematoxylin and eosin (H&E) staining as described in the methods. Figure 1 C shows H&E staining of FETα and FETα-DN primary tumors; as well as metastatic colonies in the lungs of FETα-DN orthotopically implanted animals. These results indicate that while FETα and FETα-DN cells are both 100% invasive at the primary implanted site, however, there was a robust increase observed in metastatic potential after removal of TGFβ signaling by DNRII. FETα bearing animals had a 5% metastatic rate compared to a 77% metastatic rate observed in FETα-DN bearing animals for equally sized primary tumors using previously described histological assessment methodology [42].

**Table 1:** FETα implant develop primary invasion but no metastasis

		
**Implant**	**Primary Invasion** 44/44****	**Metastasis** 2/44****
***FET α***	**(100%)**	**(5%)**

**Table 2:** Loss of TGFβ tumor suppressor activity results in robust metastasis

		
**Implant**	**Primary Invasion 30/30**	**Metastasis 23/30**
**FET α-DN**	**(100%)**	**(77%)**

**Figure 2:**
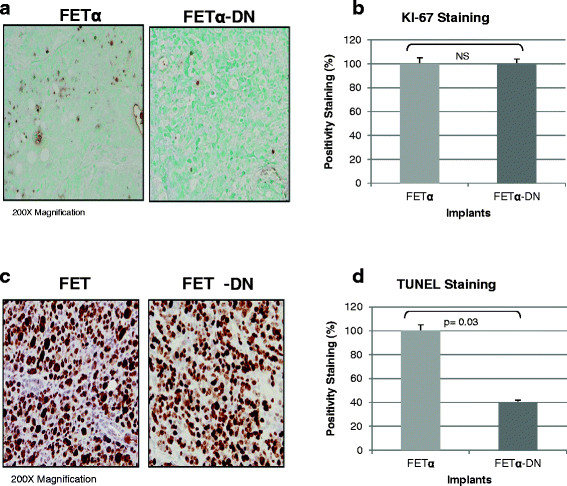
**Enhanced cell survival capability of FETα-DN cells results in metastasis.** Primary tumors established in animals orthotopically implanted with FETα and FETα-DN tumors were processed for **(A-B)** KI-67 staining and were analyzed to evaluate the proliferation rate. Primary tumors established in animals orthotopically implanted with FETα and FETα-DN tumors were processed for **(C-D)** TUNEL staining and were analyzed to evaluate the apoptotic rate. Both KI-67 and TUNEL images were captured at 200x magnification. Image J software was employed to quantify positive staining cells and the total number of cells. Statistical significance was determined using two tailed student’s *t* test with p value less than 0.05.

The increased metastatic capability of FETα-DN implants suggests that these cells acquired enhanced survival capabilities enabling them to escape from the primary tumor site to form colonies at a distal organ site as a result of loss of TGFβ inhibitory signaling. Proliferative potential and survival signaling were assessed *in situ* by KI-67 [43] and TUNEL assays [44] as previously described [42]. Immunohistochemical staining of KI-67 showed that both FETα and FETα-DN tumors had positive staining for KI-67 antigen. KI-67 staining indicated no differences in the proliferation rates between FETα and FETα-DN implanted animals (Figure 2A and 2B). However, TUNEL staining was higher in tumors from FETα implanted animals thus, reflecting a larger number of cells undergoing apoptosis in FETα tumors as compared to FETα-DN tumors (Figure 2C). The apoptotic rate of FETα implants was 2.5-fold that of FETα-DN implants (Figure 2D). Taken together these results indicate that the level of TGFβ receptor/Smad signaling in FETα cells is not capable of suppressing tumor initiation and invasion, but does suppress the progression of a primary invasive carcinoma to a robust metastatic capability. Thus, shifting the tumor suppressor/oncogenic balance toward oncogenesis by constitutive EGFR activation allows for malignancy, but not a robust metastatic phenotype due to continued metastasis suppressor signaling by TGFβ.

### Abrogation of TGFβ tumor suppressor signaling *in vitro* results in enhanced survival during GFDS

**Figure 3:**
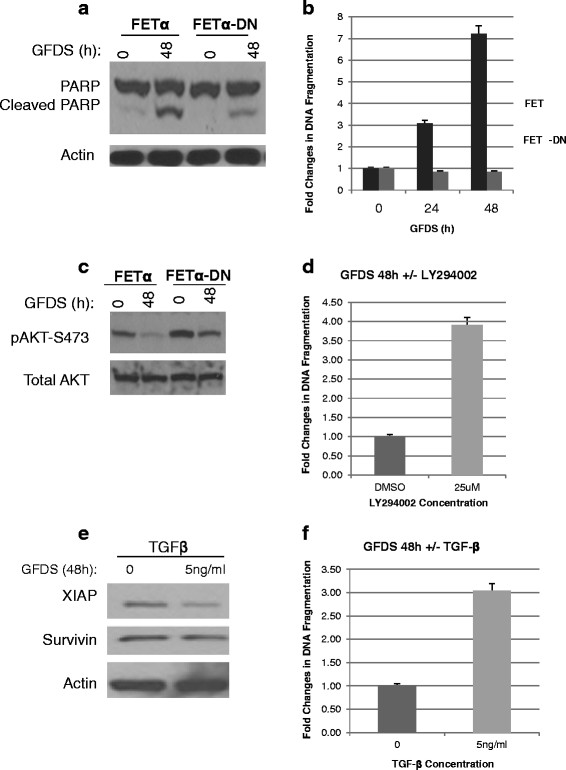
**Evidence for enhanced cell survival *in vitro*.** FETα and FETα-DN cells were grown to 80% confluence followed by growth factor deprivation for 48 h and probed for differences in apoptosis as reflected by several approaches. **(A)** Western blot analysis was performed for PARP and cleaved PARP antibodies, using actin as a loading control. **(B)** DNA fragmentation assay was utilized to determine apoptosis. *Error bars* represent S.E. **(C)** Western blot analysis was performed for AKT phosphorylation at S473, using total AKT as the experimental control. **(D)** DNA fragmentation assay of LY294002 (25uM) treated FETα cells under GFDS conditions described in the Material and Methods. *Error bars* represent S.E. FETα cells were grown to 80% confluence followed by deprivation of growth factors in the absence or presence of 5 ng/ml TGFβ for 48 h. Assessment of apoptotic activity of exogenous TGFβ was determined **(E)** Western blot analysis probing for XIAP and survivin, using actin as a loading control. **(F)** DNA fragmentation assay. *Error bars* represent S.E.

The ability of FETα cells to carry out invasion at the primary site, but not carry out subsequent aspects of the metastatic cascade due to TGFβ signaling suggests that this tumor suppressor activity is strong enough to shift the balance of tumor suppressor/oncogenesis signaling toward cell death when these cells encounter the stresses associated with various steps that must be traversed in the metastatic process such as circulation in the blood and/or colonization in the foreign microenvironment of distant organs (reviewed by Mehlen and Puisieux [4]). The loss of TGFβ associated tumor suppressor activity would be expected to shift this balance towards a higher capacity for cell survival in the FETα-DN cells. To test the hypothesis that loss of TGFβ tumor suppressor signaling resulted in a higher capacity for cell survival, we utilized growth factor deprivation as a cell survival stress model to compare FETα and FETα-DN cells as previously described for FET cells [16]. Cells were deprived of growth factors for 48 h followed by determination of apoptosis. Assessment for apoptotic behavior was performed by immunoblot analysis probing for poly-(ADP-ribose) polymerase (PARP) expression and cleavage. The appearance of cleaved products of PARP (88 kDa) has been widely used as an indicator of apoptosis [45]. Immunoblot analysis was used to probe for PARP following 48 hrs GFDS. Figure 3A illustrates that PARP cleavage is robust in FETα cells deprived of growth factors for 48 h while PARP cleavage in FETα-DN cells is low. Additional confirmation of an enhanced survival phenotype in FETα-DN cells was obtained by DNA fragmentation analysis after 48 hrs GFDS. Figure 3B indicates that FETα cells had a time dependent increase in DNA fragmentation during GFDS (up to 7-fold at 48 h) as compared to FETα-DN cells. Taken together, these results indicate that endogenous TGFβ signaling is responsible for a high level of apoptotic signaling in FETα cells as abrogation of TGFβ inhibitory signaling in the FETα-DN cells rendered the cells more resistant to apoptosis.

### Increased AKT activation and survivin/XIAP expression through repression of TGFβ signaling contributes to cell survival

Based on our previous observation that endogenous TGFβ signaling repressed PI3K/AKT signaling in tissue culture and that this repression was critical to induction of apoptosis in stressed FET cells [16], we determined whether PI3K/AKT activation by repression of TGFβ signaling contributed to the enhanced cell survival that resulted from loss of TGFβ inhibitory signaling in FETα-DN cells using pAKT modulation as an indicator of PI3K/AKT signaling. Cells were grown to 80% confluence and deprived of growth factors for 48 h then subjected to immunoblot analysis for AKT phosphorylation. The results showed that phosphorylation of AKT was decreased in FETα cells relative to FETα-DN cells under both GFDS stress and normal growth conditions (Figure 3C). To confirm that PI3K/AKT signaling was linked to cell survival in FETα-DN cells we treated cells with LY294002, a potent inhibitor of PI3K. The effect of LY294002 inhibition on cell survival was determined by growing cells to 80% confluence followed by growth factor deprivation for 48 h in the presence of DMSO or 25 uM LY294002. Confirmation of inhibition of apoptosis was assessed by DNA fragmentation analysis. Results demonstrated that LY294002 treated FETα-DN cells had a 4 fold increase in apoptosis compared to DMSO treated cells (Figure 3D).

Survivin has been implicated in aberrant cell survival exhibited by tumorigenic cells [24]. AKT mediated phosphorylation of XIAP within the Bir1 domain has been shown to reduce ubiquitination of this protein and thus enhance its stabilization [29]. There is evidence indicating that XIAP is stabilized through its interaction with survivin [27]. Survivin protects XIAP from proteasomal degradation and antagonizes apoptosome-mediated cell death through the ability of XIAP to inhibit caspase activation [26]. Consequently, we hypothesized inhibition of TGFβ signaling would also enhance expression of both survivin and XIAP. Cells were grown to 80% confluence then treated with 5 ng/mL TGFβ in combination with GFDS for 48 h followed by immunoblot analysis for survivin, XIAP and actin. As shown in Figure 3E, exogenous TGFβ inhibited survivin and XIAP expression in stressed FETα cells. To assess the effect of TGFβ treatment on cell survival, cells were treated in the presence or absence of 5 ng/mL TGFβ in combination with GFDS for 48 h followed by DNA fragmentation assays which showed a 3-fold increase in DNA fragmentation of FETα cells treated with TGFβ (Figure 3F). These results indicate that TGFβ mediated inhibition of survivin and XIAP expression is associated with FETα cell sensitivity to apoptosis*.*

**Figure 4:**
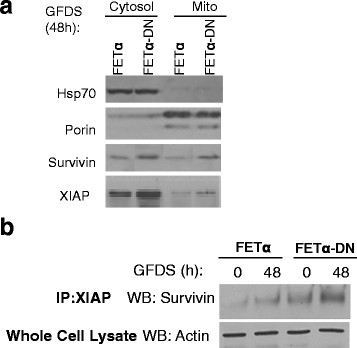
**Determination of the localization and interaction of molecular markers *in vitro*.** FETα and FETα-DN cells were grown to 80% confluence followed by growth factor deprivation for 48 h. **(A)** Subcellular fractionation to separate cytosolic from mitochondrial fractions was performed on FETα and FETα-DN cells followed by western blot analysis probing for XIAP and survivin. Hsp70 and porin proteins were used as compartmentalization controls for cytosol and mitochondria, respectively; or **(B)** Differences in survivin/XIAP complex formation were determined by immunoprecipitation with XIAP mouse monoclonal antibody followed by western blot analysis was performed for survivin.

Localization of survivin plays a major role in its function. Survivin can be nuclear, mitochondrial, cytoplasmic or associated with the mitotic apparatus [46]. It has been reported that tumor cells have high levels of survivin in the mitochondria that are released into the cytosol upon stress stimulation to provide a cytoprotective effect [26]. Cytoplasmic survivin binds XIAP and enhances XIAP stability by protecting it from proteasomal degradation and antagonizes apoptosome-mediated cell death through the ability of XIAP to inhibit caspase activation *in vivo*[26]. Therefore we hypothesized that abrogation of TGFβ signaling resulted in enhanced expression of survivin and XIAP in the cytoplasm. To test this hypothesis we performed subcellular fractionation to interrogate survivin and XIAP localization in FETα versus FETα-DN cells *in vitro.* The rationale for separation of the cytosolic and mitochondrial fractions was to assess whether there were differences in the cytoplasmic pools of survivin between the cell lines that correlates with the enhanced cell survival signaling observed in FETα-DN cells. Porin was used as a mitochondrial specific control, while Hsp70 was used as a cytosolic compartment marker. Figure 4A shows that FETα-DN cells have more cytosolic and mitochondrial survivin and XIAP, while no difference was observed in nuclear survivin (data not shown). Immunoprecipitation (IP)/western blot analysis was performed to confirm that the stabilizing complex formation by these two proteins was more prominent in FETα-DN cells. Figure 4B shows FETα and FETα-DN lysates subjected to IP for XIAP followed by immunoblot analysis of survivin, while the supernatant was probed for actin as an experimental control. Our results showed that FETα-DN cells have greater complex formation between XIAP and survivin proteins under stress conditions than FETα cells.

**Figure 5:**
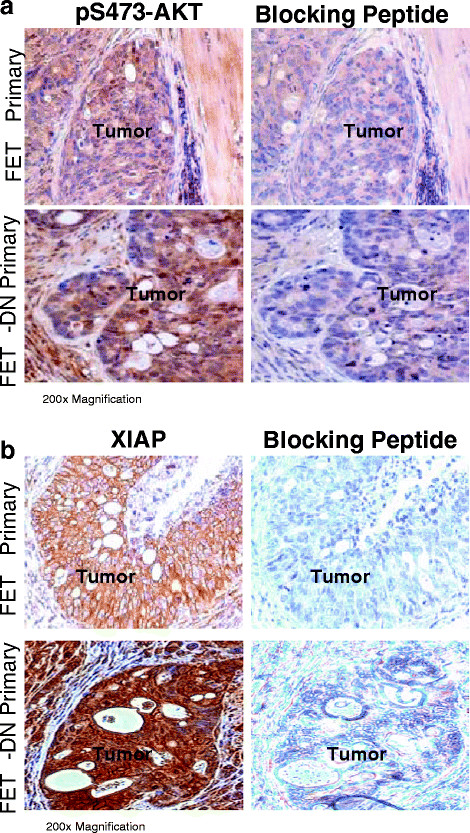
**Determination of the status of molecular markers *in vivo*. (A)** Immunohistochemical staining of pAKT-S473 performed on FETα and FETα-DN primary tumors. To confirm antibody specificity, a blocking peptide was used. **(B)** Immunohistochemical staining of XIAP protein performed on FETα and FETα-DN primary tumors. To confirm antibody specificity, a blocking peptide was used.

To ascertain whether enhanced *in vitro* molecular marker expression was also reflected *in vivo*, immunohistochemical staining (IHC) using specific antibodies for pAKT and XIAP was employed to stain tissue sections of orthotopic implants. IHC staining of FETα and FETα-DN orthotopic implants was performed with a phosphospecific AKT S473 antibody. A blocking peptide that corresponds to the same epitope as the antibody was used as a negative control. Not surprisingly, AKT activation was visible in both FETα and FETα-DN histological slides processed simultaneously. However, the intensity of staining was more pronounced in the FETα-DN implants (Figure 5A). To determine whether differences in XIAP expression were associated with cell survival, IHC staining was performed on FETα and FETα-DN implants with a XIAP specific antibody and a specific blocking peptide control (Figure 5B). Our results showed that FETα-DN implants had stronger staining for XIAP as compared to FETα implants. Collectively, these results indicate that TGFβ signaling leads to repression of activated AKT and XIAP expression, and their subsequent association with cell survival.

### Restoration of TGFβ signaling to native cells with compromised TGFβ signaling suppressed cell survival and metastasis *in vivo*

**Figure 6:**
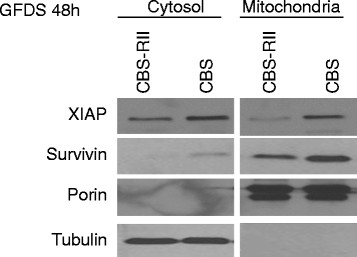
**Restoration of TGF**β **signaling suppresses enhanced cell survival signaling *in vitro*.** The native CBS cell line (low expression of TGFβRII receptor) and CBS-RII (transfected with TGFβRII) were utilized to demonstrate that re-introduction of TGFβ signaling results in suppression of cell survival signaling. CBS and CBS-RII cells were grown to 80% confluence, and subjected to GFDS for 48 h followed by subcellular fractionation to separate cytosolic from mitochondrial fractions. Western blot analysis was used to probe XIAP and survivin. Tubulin and porin proteins were used as compartmentalization controls for cytosol and mitochondria, respectively.

Up to this point demonstration of TGFβ mediated suppression of metastases was based on genetic blockade of TGFβ signaling. We extended these results by using the reverse strategy in which metastatic capability in native colon carcinoma cells was reversed through introduction of TGFβ receptor Smad signaling. CBS is a human colon carcinoma cell line that has attenuated TGFβ signaling as a result of reduced expression of TGFβ receptor type II [38]. TGFβ sensitivity was restored to native CBS cells through stable reintroduction of TGFβ type II receptor (designated CBS-RII). Subcellular fractionation was performed on CBS and CBS-RII cells to determine whether restoration of TGFβ receptor signaling resulted in suppression of survivin/XIAP expression. CBS-RII cells exhibited reduced survivin and XIAP expression as compared with CBS cells *in vitro* (Figure 6). Porin was used as a mitochondrial specific control, while tubulin was used as a cytosolic compartment marker. Reintroduction of Smad-dependent TGFβ signaling resulted in decreased expression of cytoplasmic survivin and XIAP in CBS-RII cells.

**Figure 7:**
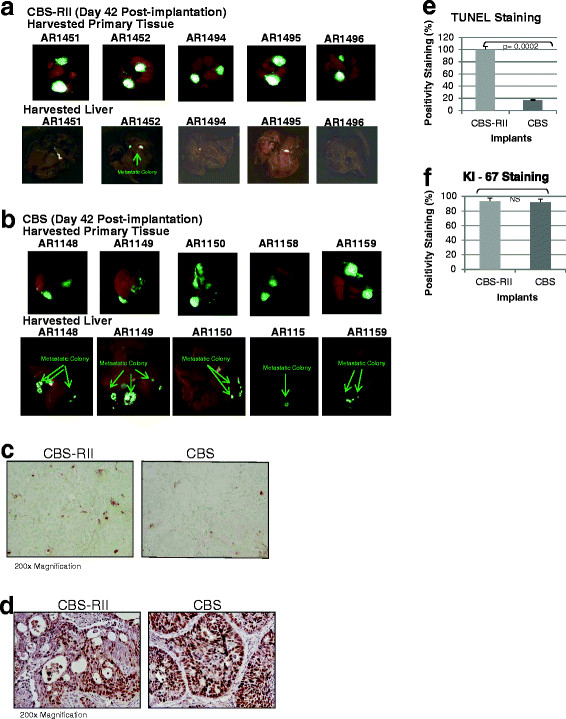
**Metastasis is suppressed through reconstitution of TGF**β **signaling. (A)** Fluorescent images of primary tumors and the liver from CBS-RII orthotopically implanted animals. Green fluorescence reflects metastatic colony formation from the primary tumor. **(B)** Fluorescent images of primary tumors and the liver from CBS orthotopically implanted animals. Green fluorescence reflects robust metastatic colony formation from the primary tumor. **(C-D)** Primary tumors established in animals orthotopically implanted with CBS-RII and CBS tumors were processed for KI-67 and TUNEL staining. Both KI-67 and TUNEL images were captured at 200x magnification. (E-F) Primary tumors formed by CBS-RII and CBS cells were analyzed to evaluate the proliferation and apoptotic rates. Image J software was employed to quantify positive staining cells and total number of cells. Statistical significance was determined using two tailed student’s *t* test with p value less than 0.0002.

To determine if reintroduction of TGFβ signaling to the CBS cells would affect their metastatic capability, we performed orthotopic implantation experiments. Figure 7A and 7B compares GFP fluorescence of the primary cancers and liver isolated from animals orthotopically implanted with CBS-RII or CBS cells, respectively. The results show that liver from CBS bearing animals had significantly more metastatic colony formation as reflected by green fluorescence, while CBS-RII bearing animals had less. Note that the yellow fluorescence comes from the biliary tree. Table 3 summarizes the results of implantation with xenografts formed by CBS-RII and CBS cells. The lungs and liver of each animal were sequentially sectioned at 1-mm intervals. We observed 100% invasiveness at the primary tumor site for both cell types. However, distal metastatic colony formation was notably different between CBS and CBS-RII animals, in which 2/20 CBS-RII bearing animals compared to 17/26 CBS bearing animals showed distal metastases. CBS-RII bearing animals had a 10% metastatic rate compared to a 65% metastatic rate observed in CBS bearing animals for equally sized primary tumors reflected by a robust reduction of the percentage of animals bearing microscopic metastases using histological assessment methodology previously described [42]. Ectopic expression of TGFβRII in CBS-RII cells did not prevent primary invasive tumor formation but did suppress the metastatic phenotype. Proliferative potential and survival signaling were assessed *in situ* by KI-67 and TUNEL assays as previously described. Immunohistochemical staining of KI-67 showed that both CBS-RII and CBS tumors had positive staining for KI-67 antigen. KI-67 staining indicated no differences in the proliferation rates between CBS-RII and CBS implanted animals (Figure 7D and 7F). However, TUNEL staining was higher in tumors from CBS-RII implanted animals thus, reflecting a larger number of cells undergoing apoptosis in CBS-RII tumors as compared to CBS tumors (Figure 7C). The apoptotic rate of CBS-RII implants was 5-fold that of CBS implants (Figure 7E). Taken together these results indicate that restoration of TGFβ receptor/Smad signaling in CBS-RII cells is not capable of suppressing tumor initiation and invasion, but does suppress the progression of a primary invasive carcinoma to a robust metastatic capability.

**Table 3:** Restoration of TGFβ tumor suppressor activity suppresses metastasis

		
**Implant**	**Primary Invasion20/20**	**Metastasis 2/20**
**CBS-*RII***	**(100%)**	**(10%)**
	**Primary Invasion26/26**	**Metastasis 17/26**
	**(100%)**	**(65%)**

## Discussion

TGFβ primes breast cancer cells for metastasis to the lung through effects on cells in the lung microenvironment [47]. Similarly, TGFβ interacts with the bone microenvironment to enhance breast cancer metastasis [47,48]. Our results show a novel role for TGFβ signaling in human colon carcinoma, as a direct metastatic suppressor through inhibition of cell survival despite acquisition of malignancy as defined by invasiveness in primary cancer cells with low metastatic potential. The mechanism of this pro-apoptotic effect appears to involve inhibition of XIAP mediated cell survival mechanisms. FETα cells have aberrant EGFR activation via TGFα over-expression resulting in formation of invasive primary colon cancer (Figure 1A), but have poor potential for forming distal organ metastasis, due to sensitivity to their intrinsic apoptotic TGFβ signaling, as shown by high levels of metastatic colonies when TGFβ signaling was blocked in FETα-DN cells (Figure 1B). We have shown that primary tumor formation is linked to enhanced cell survival mechanisms exhibited by these cells [33]. The importance of cell survival is further emphasized by the observation that abrogation of TGFβ signaling in the FETα-DN cells does not affect invasion at the primary site but facilitates secondary site colonization.

The metastatic process is complex and has multiple mechanisms that must be acquired by tumor cells before they obtain a robust metastatic capability. Two important rate limiting steps to metastasis are invasion and distal colony formation. There are few *in vivo* model systems that enable the study of both invasion and distal colony formation. We have utilized an orthotopic implantation model of colon cancer to allow observation of these events. The orthotopic implantation model allows for assessment of the progression of colon cancer evident by invasion at the primary tumor site and distal colonization to the liver and lungs. These sites of metastasis recapitulate the natural progression of human disease. Our results show that both FETα and FETα-DN cells were able to invade the bowel wall and the normal colon crypts to form a carcinoma. However, the orthotopic implants showed that the FETα-DN cells with abrogated TGFβ signaling were able to effectively generate colonies despite the stress of growth in the foreign microenvironment of distal organs, emphasizing the role of TGFβ as a metastasis suppressor as well as a tumor suppressor.

The reconstitution of TGFβ receptor signaling in CBS-RII cells resulted in decreased metastases indicating the potential for treatment of metastasis through enhanced TGFβ receptor mediated signaling. The balance between oncogenes and tumor suppressor activities is a necessity for normal functioning cells and tissues; however, when the balance shifts towards oncogenicity it results in tumorigenesis and malignant progression. CBS cells have been shown to be similar to the FETα engineered cells in that they have constitutive EGFR activation in addition to the attenuation of TGFβ tumor suppressor activity [38,49], thus providing a mechanism for retention of the capability of forming an invasive cancer at the primary site despite TGFβ activity generated by ectopic expression of the TGFβRII.

Activation of inappropriate survival mechanisms such as survivin/XIAP and/or inactivation of tumor suppressors (i.e., TGFβ) are involved in promoting cell survival during tumorigenicity and metastasis. The ability of malignant cells to withstand environmental stress is considered an important factor in tumor development and progression [1] as well as in the metastatic process [4]. Loss of TGFβ-mediated apoptosis may contribute to tumor progression and metastasis under such stress conditions. Mehlen and Puisieux [4] and Giampieri et al., [50] have reviewed the particular importance of aberrant cell survival in the establishment of metastatic colonies in the foreign microenvironment of organs distal to the primary tumor site. Moreover, different stages of the metastatic process show different mechanisms for aberrant survival. We have shown that abrogation of autocrine TGFβ enables increased PI3K/AKT activation in FETα-DN cells under GFDS, which shifts the balance of signaling during stress by these cells from apoptosis to survival thus contributing to resistance to stress induced apoptosis.

The significance of survivin subcellular localization in cell survival has been addressed by the Altieri laboratory [46]. Nuclear survivin is associated with proliferation while cytoplasmic survivin is associated with cell survival [51]. Survivin associates with another IAP family member, XIAP, in response to cell death stimuli [27]. The resultant survivin-XIAP complex promotes increased XIAP stability from ubiquitination and subsequent proteosomal degradation [26]. Tumor cells have high pools of survivin present between the mitochondrial membranes that are released into the cytosol upon stress stimulation [28]. It was shown that when cytoplasmic survivin is not phosphorylated at S20 it binds XIAP and enhances XIAP stability by protecting it from proteasomal degradation thus enabling antagonization of apoptosome-mediated cell death through the ability of XIAP to inhibit caspase −3, -7 and −9 activation *in vivo*[26]. A recent study has documented that nuclear survivin has reduced stability and is not cytoprotective [52]. Our study shows for the first time that abrogation of TGFβ signaling results in enhanced cytosolic localization of survivin and XIAP proteins which are associated with enhanced cell survival capability and eventual metastasis in the FET colon cancer cell model (Figure 4A). This observation was further validated by restoring TGFβ sensitivity in the native CBS colon carcinoma cell line (Figure 6).

We have utilized genetic modification of TGFβ receptors to show that TGFβ receptor mediated signaling is critical to the suppression of metastasis in the FET and CBS colon cancer models. The question arises as to the potential breadth of cancers in which TGFβ receptor modulation would be a factor and whether pharmacological modulation would be possible. Over the past 15 years we and others have shown that transcriptional repression of either RI or RII is seen in a variety of histological types of cancer including colon, breast, lung and pancreatic cells lines [7-9,53-56]. Along this line, several clinical studies have indicated that cancer progression is associated with loss of TGFβ receptors in types of cancers where TGFβ mutation is rare or in the case of colon cancer, in patient samples without microsatellite instability thereby implying a lack of mutation [57-62]. More recently, we have shown that cancer cell lines with TGFβ receptor repression due to histone acetylation can be rescued by treatment with a clinical HDAC inhibitor candidate. Importantly, this pharmacological rescue results in TGFβ signaling dependent induction of apoptosis through disruption of survivin/XIAP mediated cell survival as seen both *in vitro* and *in vivo* in the 2 cell lines studied here as well as a pancreatic cancer cell line and 3 breast cancer cell lines [63]. Consequently, based on the broad range of cell lines showing TGFβ receptor repression, the clinical studies of cancer progression related to TGFβ receptor loss in cancers that rarely show TGFβ receptor mutations and the pharmacological responses of cell lines demonstrating TGFβ receptor transcriptional repression, the subset of cancers in which TGFβ receptor signaling potentially enables metastasis appears to be significant in a subset of cancers. Moreover, the mechanism of TGFβ receptor repression may be susceptible to pharmacological intervention [63].

This dichotomous role of TGFβ signaling with respect to tumor progression is problematic for strategies to target aberrant TGFβ signaling in cancer. The observations presented here raise the concern that abrogation of TGFβ signaling may lead to acceleration of malignant progression even in the biological context of invasive cancer. However, reconstitution of deficient TGFβ signaling can result in the direct activation of cell death and inhibition of metastasis thus indicating TGFβ is a metastatic suppressor in fully invasive carcinomas, thus indicating that at least in some cancer contexts the concept of enhancing TGFβ activity and/or the mechanisms by which TGFβ generates cell death could be of therapeutic value in highly progressed cancers.

## Conclusion

The observations presented here indicate a metastasis suppressor role for TGFβ signaling in human colon cancer cells. This raises the concern that therapies targeting inhibition of TGFβ signaling may be imprudent in some patient populations with residual TGFβ tumor suppressor activity where consideration of enhancement of TGFβ signaling may be beneficial.

## Competing interests

The authors declare that they have no competing interest.

## Author's contribution

NS involved in experimental design, performed *in vitro* assays and IHC assays and drafted manuscript. AR performed *in vivo* orthotopic implantation experiments. ES performed histological slide preparation, histology assays and statistical analysis. MO performed *in vivo* orthotopic implantation experiments. CT performed tissue culture. JW participated in experimental design and data interpretation. MGB involved in experimental design, data interpretation, manuscript revision. All authors read and approved the final manuscript.

## Pre-publication history

The pre-publication history for this paper can be accessed here:

http://www.biomedcentral.com/1471-2407/12/221/prepub

## Supplementary Material

Additional file 1 **Abrogation of TGFβ signaling.** (a) FETα and FETα-DN cells in log phase growth were treated with varying concentrations of TGFβ [0, 5, 10 ng/mL] for 2 h followed by immunoblot analysis performed for pSmad2 and total Smad2 used as a loading control. (B) FETα and FETα-DN cells in log phase growth were treated with 0 or 5 ng/mL TGFβ for 48 hours followed by [^3^ H] thymidine labeling to assess growth inhibition. Click here for file
