# Supplementary figures and images for: Transforming growth factor-β suppresses metastasis in a subset of human colon carcinoma cells
Source: BMC Cancer. 2012 Jun 6;12:221. doi: 10.1186/1471-2407-12-221 (PMC3517326; doi:10.1186/1471-2407-12-221)

## Slide 1
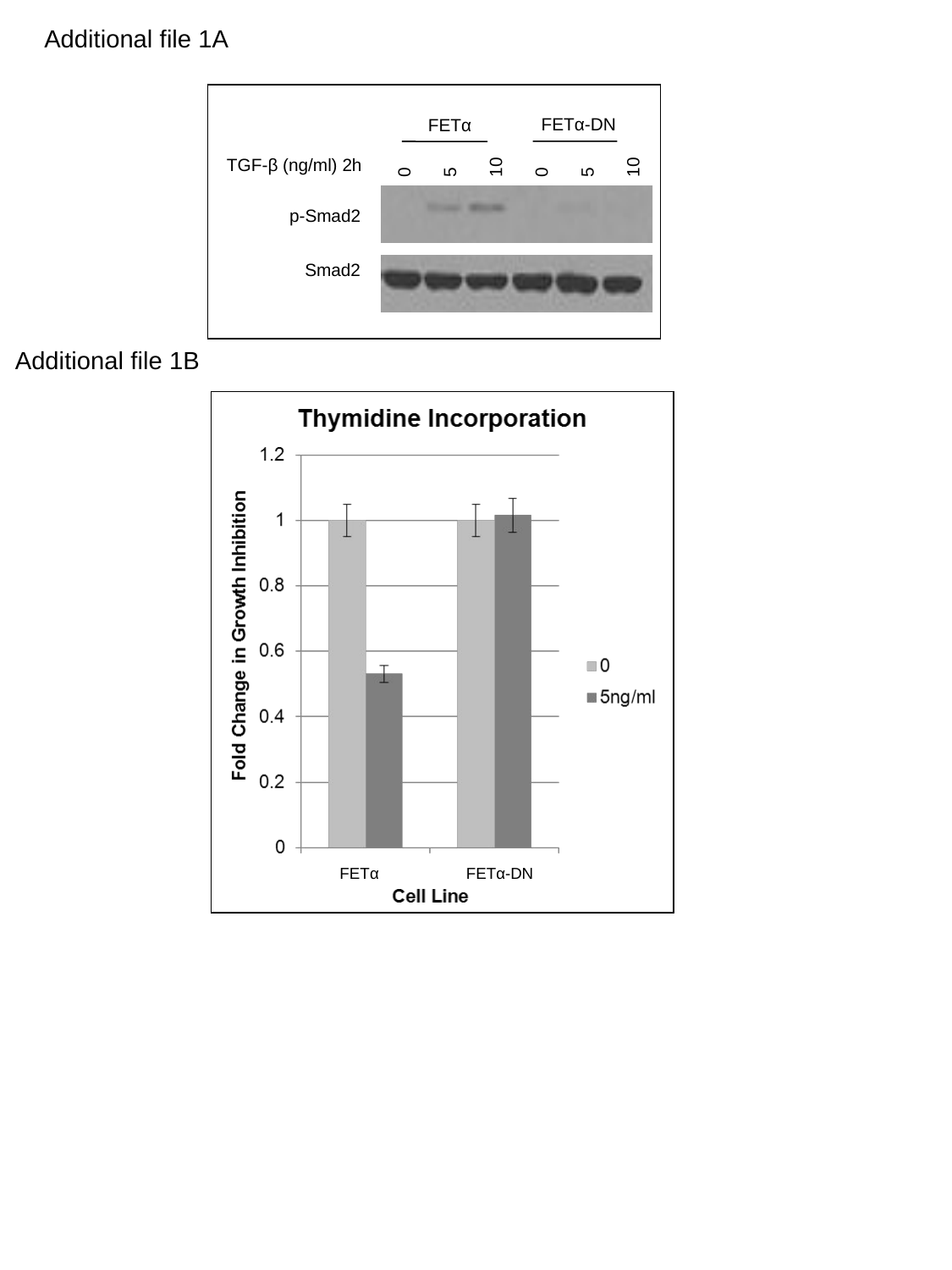

Additional file 1A
0
5
10
0
5
10
FETα-DN
FETα
TGF-β (ng/ml) 2h
p-Smad2
Smad2
Additional file 1B
FETα
FETα-DN

Supplement: Additional file 1 — Abrogation of TGFβ signaling. (a) FETα and FETα-DN cells in log phase growth were treated with varying concentrations of TGFβ [0, 5, 10 ng/mL] for 2 h followed by immunoblot analysis performed for pSmad2 and total Smad2 used as a loading control. (B) FETα and FETα-DN cells in log phase growth were treated with 0 or 5 ng/mL TGFβ for 48 hours followed by [3 H] thymidine labeling to assess growth inhibition. [file 1471-2407-12-221-S1.ppt]
